# Supplementary material for: Saccharomyces boulardii (CNCM I-745) improves intestinal damage in sepsis by remodeling intestinal flora structure
Source: Front Cell Infect Microbiol. 2025 Jul 31;15:1602792. doi: 10.3389/fcimb.2025.1602792 (PMC12350329; doi:10.3389/fcimb.2025.1602792)
Supplement: Supplementary file 1 [file DataSheet1.zip › summary/summary/7_Differential_analysis/1_diff_abundance/6_Genus/Group.AW4_vs_AW0/Group.AW4_vs_AW0_diff_barplot.pdf]

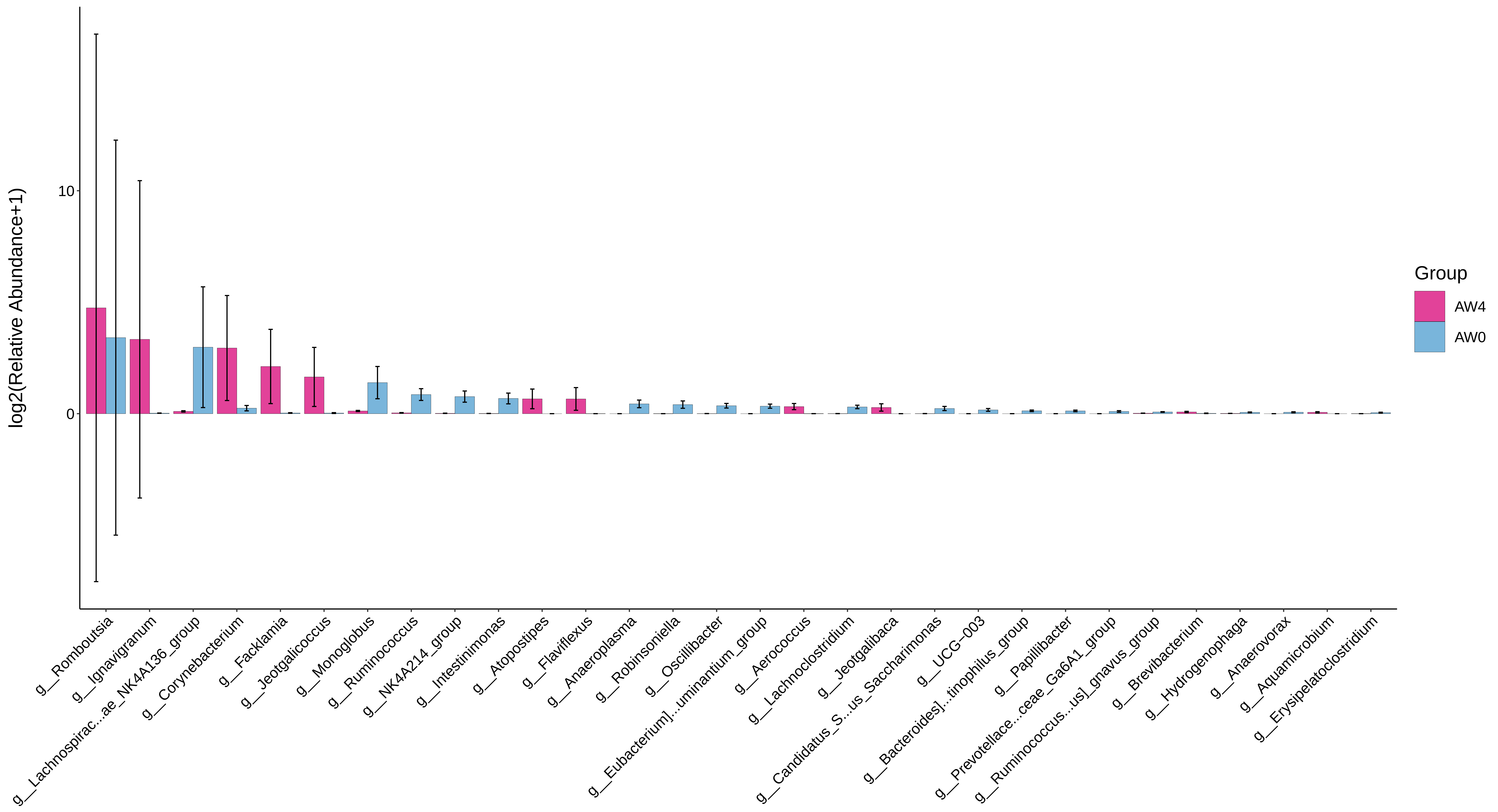

log2(Relative Abundance+1)
10
0
Group
AW4
AW0
g__Romboutsia
g__Ignavigranum
g__Lachnospirac...ae_NK4A136_group
g__Corynebacterium
g__Facklamia
g__Jeotgalicoccus
g__Monoglobus
g__Ruminococcus
g__NK4A214_group
g__Intestinimonas
g__Atopostipes
g__Flaviflexus
g__Anaeroplasma
g__Robinsoniella
g__Oscillibacter
g__Eubacterium]...uminantium_group
g__Aerococcus
g__Lachnoclostridium
g__Jeotgalibaca
g__Candidatus_S...us_Saccharimonas
g__UCG-003
g__Bacteroides]...tinophilus_group
g__Papillibacter
g__Prevotellace...ceae_Ga6A1_group
g__Ruminococcus...us]_gnavus_group
g__Brevibacterium
g__Hydrogenophaga
g__Anaerovorax
g__Aquamicrobium
g__Erysipelatoclostridium
